# MiR-18a and miR-18b are expressed in the stroma of oestrogen receptor alpha negative breast cancers

**DOI:** 10.1186/s12885-020-06857-7

**Published:** 2020-05-05

**Authors:** Nina Gran Egeland, Kristin Jonsdottir, Miriam Ragle Aure, Kristine Sahlberg, Vessela N. Kristensen, Deirdre Cronin-Fenton, Ivar Skaland, Einar Gudlaugsson, Jan P. A. Baak, Emiel A. M. Janssen

**Affiliations:** 1 Department of Pathology, Stavanger University Hospital, Box 8100, 4068 Stavanger, Norway; 2 Department of Chemistry, Bioscience and Environmental Engineering, University of Stavanger, Stavanger, Norway; 3 Department of Cancer Genetics, Institute for Cancer Research, Oslo University Hospital, Oslo, Norway; 4 Department of Research and Innovation, Vestre Viken Hospital Trust, Drammen, Norway; 5 Department of Clinical Molecular Biology (EpiGen), Division of Medicine, Akershus University Hospital, Lørenskog, Norway; 6 Department of Clinical Epidemiology, Aarhus University, Aarhus, Denmark; 7 Dr. Med. Jan Baak AS, Tananger, Norway

**Keywords:** Breast cancer, microRNA, In situ hybridization, Tumour microenvironment, Macrophages, Tumour associated macrophages (TAM)

## Abstract

**Background:**

Previously, we have shown that miR-18a and miR-18b gene expression strongly correlates with high proliferation, oestrogen receptor -negativity (ER^−^), cytokeratin 5/6 positivity and basal-like features of breast cancer.

**Methods:**

We investigated the expression and localization of miR-18a and -18b in formalin fixed paraffin embedded (FFPE) tissue from lymph node negative breast cancers (*n* = 40), by chromogenic in situ hybridization (CISH). The expression level and in situ localization of miR-18a and -18b was assessed with respect to the presence of tumour infiltrating lymphocytes (TILs) and immunohistochemical markers for ER, CD4, CD8, CD20, CD68, CD138, PAX5 and actin. Furthermore, in two independent breast cancer cohorts (94 and 377 patients) the correlation between miR-18a and -18b expression and the relative quantification of 22 immune cell types obtained from the CIBERSORT tool was assessed.

**Results:**

CISH demonstrated distinct and specific cytoplasmic staining for both miR-18a and miR-18b, particularly in the intratumoural stroma and the stroma surrounding the tumour margin. Staining by immunohistochemistry revealed some degree of overlap of miR-18a and -18b with CD68 (monocytes/macrophages), CD138 (plasma cells) and the presence of high percentages of TILs. CIBERSORT analysis showed a strong correlation between M1-macrophages and CD4+ memory activated T-cells with mir-18a and -18b.

**Conclusions:**

Our study demonstrates that miR-18a and miR-18b expression is associated with ER- breast tumours that display a high degree of inflammation. This expression is potentially associated specifically with macrophages. These results suggest that miR-18a and miR-18b may play a role in the systemic immunological response in ER^−^ tumours.

## Background

Oestrogen receptor alpha (ER) expression is the most widely used predictive biomarker for breast cancer. Most patients with ER positive (ER^+^) tumours receive adjuvant endocrine therapy and have a good prognosis. In contrast, ER negativity (ER^−^) is found in roughly 15% [1, 2] of all breast cancers, and these tumours are often associated with high proliferation and a relatively poor prognosis. Additionally, there are few effective adjuvant therapy options for this group and for the so-called triple-negative (TNP) breast cancers that lack expression of ER, progesterone receptor (PR) and human epidermal growth factor receptor 2 (HER2). Therefore, there is a need for identifying new prognostic biomarkers and more specific, novel targets for therapy. Also, more knowledge of the biology of these tumours is necessary to improve the prognosis of patients with ER^−^ breast cancer.

MicroRNAs are defined as short non-coding RNA molecules, of which the mature form is about 22 nucleotides in length*.* Each microRNA is complementary or partially complementary to several mRNA molecules, and its main function is to post-transcriptionally down-regulate gene expression by either binding directly to its mRNA target, or by cleaving target mRNA by binding to its 3′-untranslated region (UTR) [3]. Some microRNAs are predicted to bind several hundred gene targets (mRNAs), and different microRNAs can also target the same gene [4]. Studies of mammalian cells have shown that microRNAs are one of the largest groups of translational regulators in human cells [3], and they are known to play a significant role in many cellular functions [5] and in a number of diseases, including cancer [6, 7].

Previously, we have shown that gene expression of miR-18a and miR-18b is strongly correlated with high proliferation, ER^−^ and cytokeratin 5 and − 6 positivity (CK5/6+) [8, 9]. MiR-18a belongs to the miR-17 ~ 92 cluster located on chromosome 13, while miR-18b belongs to the miR-106a ~ 363 cluster located on chromosome X [10, 11]. MiR-18a and miR-18b, and their cluster members, are mostly described as onco-microRNAs because they show higher expression in many different tumour types, and especially in more advanced tumours [10, 12]. Several studies have shown that the expression of miR-18a and miR-18b is associated with ER- status [8, 13–15], and research suggests that ER can be a direct target of miR-18a [13, 16, 17].

Besides cancer cells, tumour tissue is made up of stromal cells such as fibroblasts, adipocytes, endothelial cells and various immune cells. The tumour microenvironment (TME) contains a heterogeneous collection of immune cell types, such as T-cells and B-cells, natural killer cells, macrophages, dendritic cells and neutrophils (reviewed in [18, 19]). MicroRNAs are also involved in the interplay between cancer and immune cells [20]. It has been reported that microRNAs take part in cell-cell signalling and communication between tumour cells and the surrounding microenvironment [21], by means of paracrine signalling [22, 23] and release of extracellular vesicles [24], especially exosomes [25, 26]. It is now recognized that the TME plays a critical role in both initiation and progression of cancer, and thus has prognostic potential. The cells within the TME take part in bidirectional cross-talk and interactions with the malignant cells, and they can have pro- or anti-tumour functions, depending on the type of immune cells involved [27, 28]. Cancer-associated immune cells also play a role in treatment response [26], and may have therapeutic potential. Several studies have shown the prognostic relevance of tumour-infiltrating lymphocytes (TILs) in breast cancer, especially in the HER2-positive and triple-negative subtypes (reviewed in [29]).

Here, we applied chromogenic in situ hybridisation (CISH) and immunohistochemistry (IHC), to locate and identify which cells express miR-18a and miR-18b in breast cancer. To further investigate the origin of these cells, we applied the analytical tool CIBERSORT [30] that uses gene expression data from bulk tumour to deconvolute expression and derive relative quantification of hematopoietic cell populations, to assess which cell types miR-18a and miR-18b are associated with.

## Methods

### Patients

This study was approved by the Norwegian Regional Committees for Medical and Health Research Ethics (REC). All patients were treated according to the national guidelines of the Norwegian Breast Cancer Group (NBCG) at the time of diagnosis.

*Stavanger cohort*: Breast cancer patients diagnosed with first onset invasive operable (T_1,2_N_0_M_0_) lymph node negative breast cancer at the Stavanger University Hospital between January 1, 1993 and December 31, 1998. From this Stavanger cohort, several sub-cohorts have been used in the present study: 1) A total of 94 lymph node negative breast cancer patients from previous studies [9, 31] with complete mRNA- and microRNA expression data, hereafter called the Stavanger array-cohort, were included for correlation analysis between CIBERSORT output (based on mRNA expression) and miR-18a/miR-18b expression. 2) We analysed TILs in 204 samples (from our previous study [8]), and correlated this with our previous expression data of miR-18a and -18b (measured by quantitative real-time PCR (qPCR)), and grouped the patients based on ER status and high vs low TILs. This sub-cohort will here onwards be referred to as the Stavanger qPCR-cohort.

*CISH cohort*: Based on our previous findings of higher amounts of miR-18a and -18b in ER^−^ breast cancer, a total of 40 samples from the *Stavanger qPCR-cohort* [8] described above were selected for CISH as follows: 20 tumours classified as ER^+^ with low expression of miR-18a and miR-18b (as measured by qPCR), and 20 tumours classified as ER^−^ with high expression of miR-18a and miR-18b (as measured by qPCR). We analysed TILs in the 40 tumours and correlated this with the CISH expression of miR-18a and -18b. For the patients’ clinical characteristics, please see additional files (Additional file 1, S1 Table). Furthermore, CISH was performed also on lymph nodes histologically negative for tumour cells from two ER^−^ patients, as well as on lymph nodes histologically positive for metastasis from four ER^+^ or ER^−^ patients. Also, CISH was performed on a test block consisting of several tumour types from different patients, and on a lymph node diagnosed as reactive lymphadenitis from the neck (this patient had no history of breast cancer and had no other clinical symptoms).

*Oslo2 cohort:* a multicentre study of breast cancer patients with primary operable breast cancers consecutively enrolled from hospitals in the Oslo region from 2006 until today. Patients were included at the time of primary surgery. Tumours from the Oslo2 study (*n* = 308) and from a similar study conducted at the Akershus University Hospital (Ahus), Norway, from 2003 to 2010 (*n* = 69) were selected for CIBERSORT analyses and correlation to miRNA expression. Total RNA was isolated from fresh-frozen (Oslo2) or RNA*later*® (Ahus) material using TRIzol™, and microRNA expression data for altogether 377 tumours were correlated with matching CIBERSORT output based on mRNA expression [32, 33] using Agilent microarrays (Agilent Technologies, Santa Clara, CA, USA). For CIBERSORT analyses, both lymph node positive and -negative patients were included (*n* = 377).

### Histopathology and immunohistochemistry

The tumour tissue was fixed in buffered 4% formaldehyde and then embedded in paraffin. Sections were cut at a thickness of four μm and stained with haematoxylin, erythrosine and saffron. The histological type was assessed according to the World Health Organization criteria [34] and the tumour grade was assessed according to the Nottingham grading system [35]. ER was scored positive if ≥1% of tumour cells exhibited nuclear staining, while all others were scored negative. All sections were scored independently by two experts.

IHC was used to detect ER, CD4, CD8, CD20, CD68, CD138, PAX5 and actin. These methods were based on DAKO technology as described previously [36]. In brief, FFPE-sections of 2 μm thickness were mounted onto Superfrost Plus slides (Menzel, Braunschweig, Germany). Antigens were retrieved with a highly stabilized retrieval system (ImmunoPrep; Instrumec, Oslo, Norway) using 10 mM TRIS/1 mM EDTA (pH 9.0) as the retrieval buffer. Slides for actin staining were not treated with retrieval buffer. Sections were heated for 3 min at 110 °C followed by 10 min at 95 °C then cooled to 20 °C. The sections were incubated with monoclonal antibody at the dilutions stated in Table 1.

**Table 1:** Monoclonal antibodies used in IHC staining

**Antibody**	**Clone**	**Concentration**	**Target cells**	**Company**
ER	SP1	1:400	Epithelial cells	Thermo Scientific, Waltham, USA
CD4	4B12	1:100	CD4+ T-cells	Novocastra, Newcastle Upon Tyne, UK
CD8	C8/144B	1:50	CD8+ T-cells	DAKO, Agilent Technologies, Santa Clara, CA, USA
CD20	L26	1:1000	B-cells, neoplasms of B-cells	DAKO
CD68	PG-M1	1:400	B-cells, macrophages, histiocytes, dendritic cells, NK cells	DAKO
CD138	B-A38	1:50	Plasma cells and some epithelial cells	AbD Serotec (BioRad), Kidlington, UK
PAX5	24	1:100	B-cells	BD Biosciences, San Diego, USA
Actin	1A4	1:300	Fibroblasts	DAKO

Antibody, clone, concentrations used, Target cells and company of the antibodies used in IHC staining

### MicroRNA and mRNA expression profiling

The microRNA and mRNA expression profiling data from fresh-frozen tumour tissue used in this analysis have been published previously [9, 31–33].

### Chromogenic in situ hybridization

CISH was performed on FFPE tissue using miRCURY LNA™ microRNA ISH optimization kit (FFPE) v1.3 (Exiqon, Vedbaek, Denmark). The manufacturer’s protocol was followed with some minor changes. Briefly, 5 μm thick paraffin sections were mounted on Superfrost™ Plus glass slides and incubated overnight at 55 °C. The slides were deparaffinised with xylene and alcohol dilutions. The slides were then washed in PBS for 4 min, digested with 15 μg/ml of Proteinase K at 37 °C for 30 min, and washed in PBS before dehydration through a series of graded alcohol. The slides were hybridized with double DIG labelled Locked Nucleic Acid™ (LNA™) (Exiqon) probes at 55 °C for 1 h (see Additional file 2, S2 Table). The probe concentrations were 80 nM for the hsa-miR-18a-5p, hsa-miR-18b-5p and scramble probe, and 2.0 nM for the positive control probe U6. After hybridization the slides were washed consecutively with 5x SSC, 1x SSC and 0.2x SSC (Sigma Aldrich, St. Louis, MO, USA) at 50 °C for a total of 30 min. Then the slides were incubated with blocking solution containing 1x Maleic acid buffer (Roche, Mannheim, Germany), 10x Blocking Solution (Roche) and 2% sheep serum (Jackson Immunoresearch, Suffolk, UK) for 15 min, before application of 1:800 dilution of sheep anti-DIG alkaline phosphatase (Roche) at 30 °C for 30 min. The slides were then washed with 1% Tween-PBS for 3 × 3 min, before they were incubated with AP substrate containing NBT/BCIP (Roche) at 30 °C for 110 min. This allowed for visualization of antibody signals by NBT-BCIP. Sections were then washed twice for 5 min in KTBT buffer (50 mM Tris-HCl, 150 mM NaCl, 10 mM KCl), before being rinsed in ultrapure water 2 × 1 min. For nuclear counterstaining, the sections were immersed in Nuclear Fast Red (Sigma Aldrich, St. Louis, MI, USA) for 3 min, and then rinsed in running tap water for 5 min. Finally, the sections were dehydrated through a series of graded alcohol, and subsequently mounted by Histokitt mounting medium (VWR, Oslo, Norway).

### Quantification of miR-18a and miR-18b

Specific staining for both microRNAs was observed as a dark blue colour from the NBT/BCIP precipitation. The sections were examined by light microscopy, and positive miR-18a and miR-18b staining was quantified by cell counting in two selected areas with the highest number of positive cells within the tumour area. In these hotspot areas all positive cells were counted at 40x in an area of 1.59 mm^2^. The two areas were scored separately, and the sum of both made up the final score for each slide. Dark blue cells without a visible nucleus or distinct cell membrane were excluded, as were light purple cells. Sections with negative U6 staining or slides in which a substantial amount of material was lost during the experimental treatment, were also excluded from the study (*n* = 4).

### Scoring of lymphocyte infiltration

The variable degree of lymphocytic infiltration in HE-stained tissue sections was evaluated semi-quantitatively. First, the sections were assessed according to the presence or absence of stromal tumour infiltrating lymphocytes (sTILs). Second, the relative amount of TILs in the tumour stroma area was assessed according to the method described by Denkert et al. [37]. The degree of infiltration was scored in the range of 0–100%.

### CIBERSORT analysis

CIBERSORT (Cell-type Identification By Estimating Relative Subsets Of RNA Transcripts) is a computational framework, that on the background of gene expression data and a signature gene file quantifies the relative or absolute levels of member cell types in a mixed cell population [30]. We ran CIBERSORT using the LM22 signature gene file which provides the relative abundance of 22 distinct mature human hematopoietic populations. The mRNA expression data from the Stavanger array [31] (*n* = 94) and Oslo2 [32] (*n* = 377) cohorts were used and the maximum number of permutations (*n* = 1000) were chosen. The output from CIBERSORT was a matrix with quantification of the 22 cell types for each tumour sample.

### Statistical analyses

Statistical analyses were conducted using the software program SPSS (version 20.0, SPSS, Chicago, IL, USA) and R [38]. Differences between patient groups were tested using an independent T-test and correlations between different expression levels were done using both Pearson’s and Spearman’s correlation. To assess the association of cell type composition with miR-18a and -18b, we calculated the Spearman correlation coefficient between miR-18a and -18b expression and the CIBERSORT-based quantification of 22 hematopoietic cell types. Finally, the correlations were ranked in decreasing order.

## Results

### Expression of miR-18a and miR-18b in FFPE tissue

**Fig. 1:**
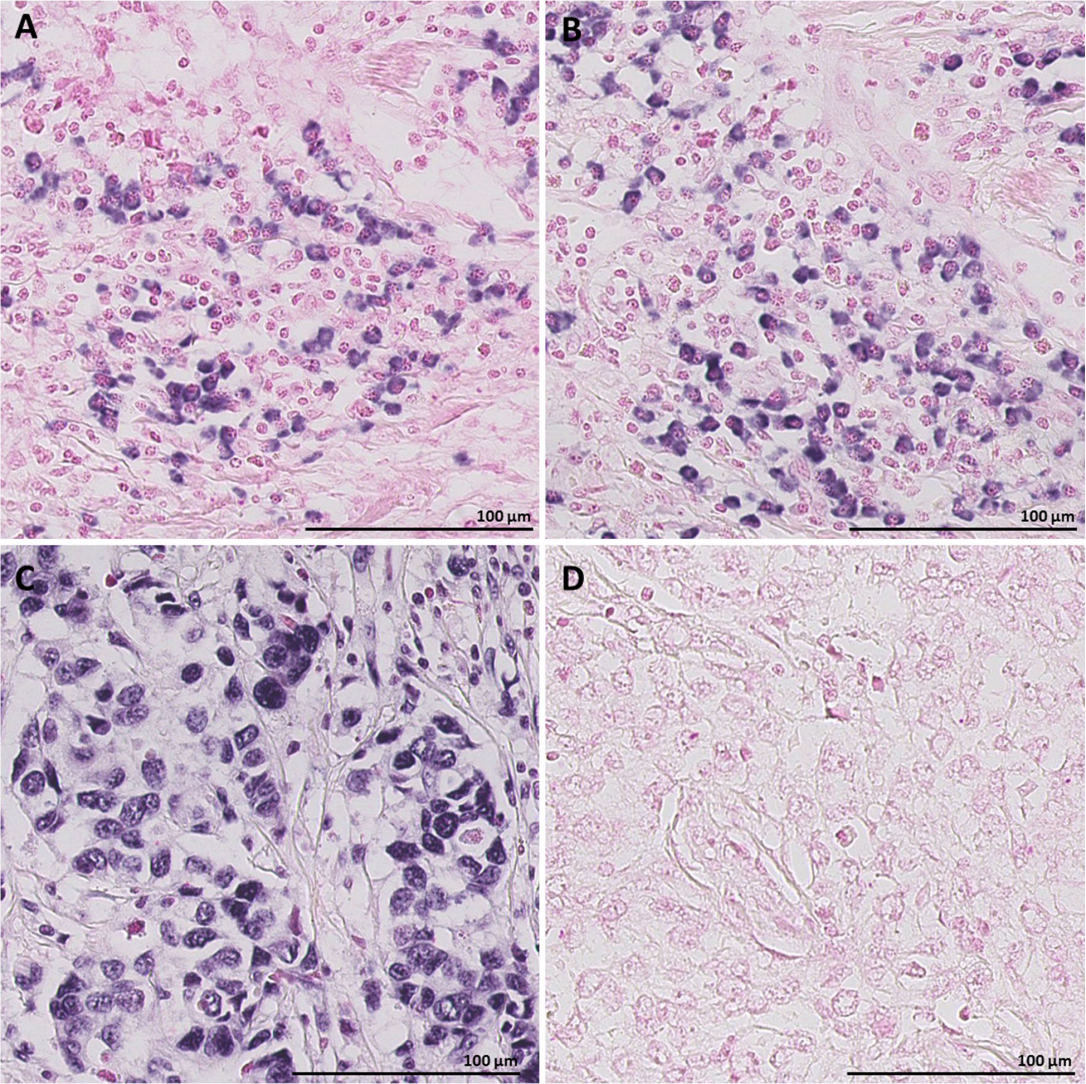
40x magnification illustrating representative CISH staining with: **a**) LNA 5`-3’DIG hsa (80 nM) miR-18a probe showing strong and specific staining in stroma, **b**) LNA 5`-3’DIG hsa (80 nM) miR18-b probe showing strong and specific staining in stroma, **c**) U6 snRNA positive control probe showing overall nuclear staining, and **d**) negative probe (scrambled) probe with no hybridization signal. Scale bar 100 μm

**Fig. 2:**
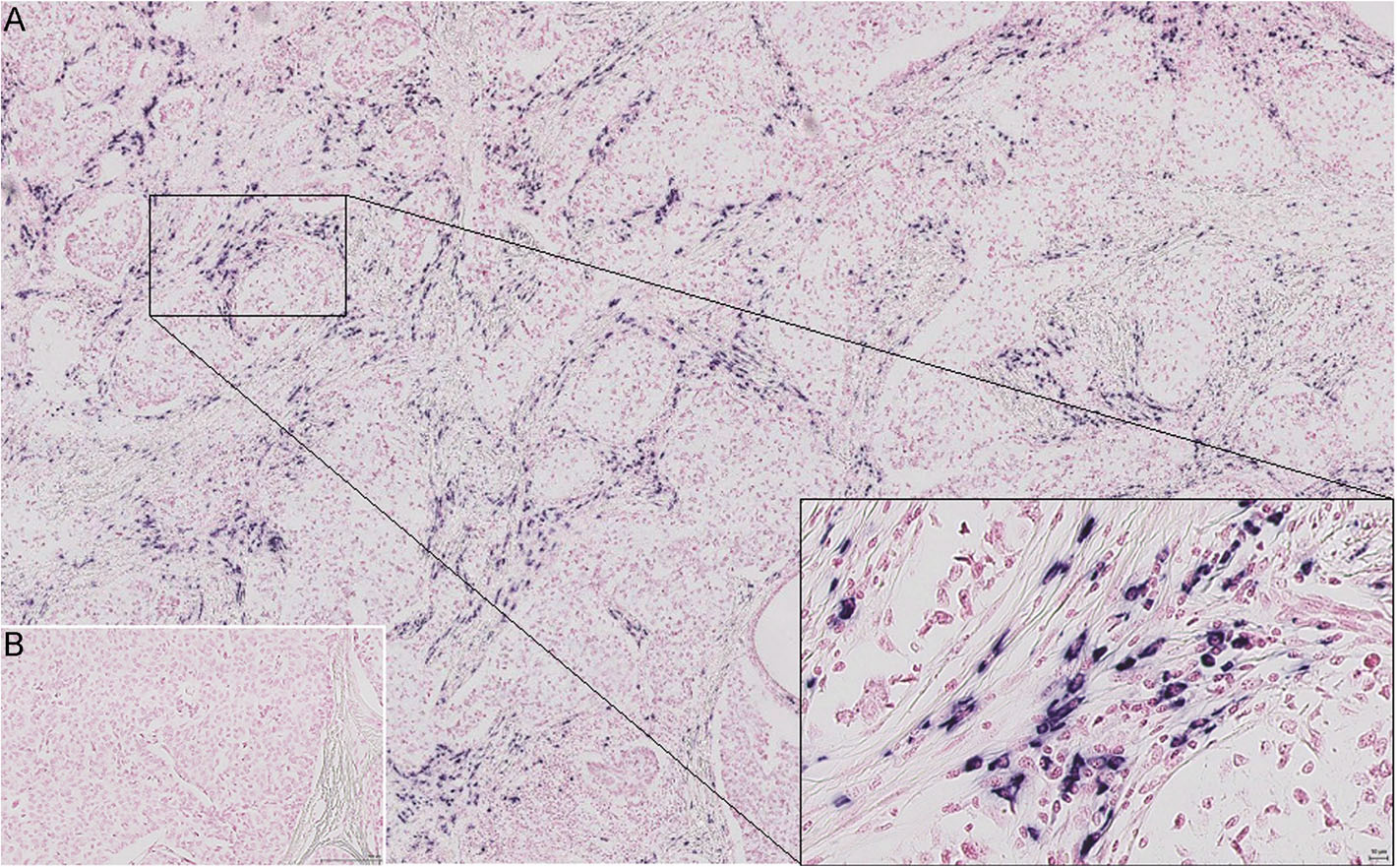
Representative miR-18b CISH expression in an ER^−^ breast tumour (sample id: 20 ER^−^ in Table 2). **a**) Specific blue (NBT/BCIP) staining for LNA 5`-3’DIG hsa miR-18b (80 nM) probe. **b**) Negative control, i.e. staining with an LNA 5`-DIG scrambled probe (80 nM)

**Fig. 3:**
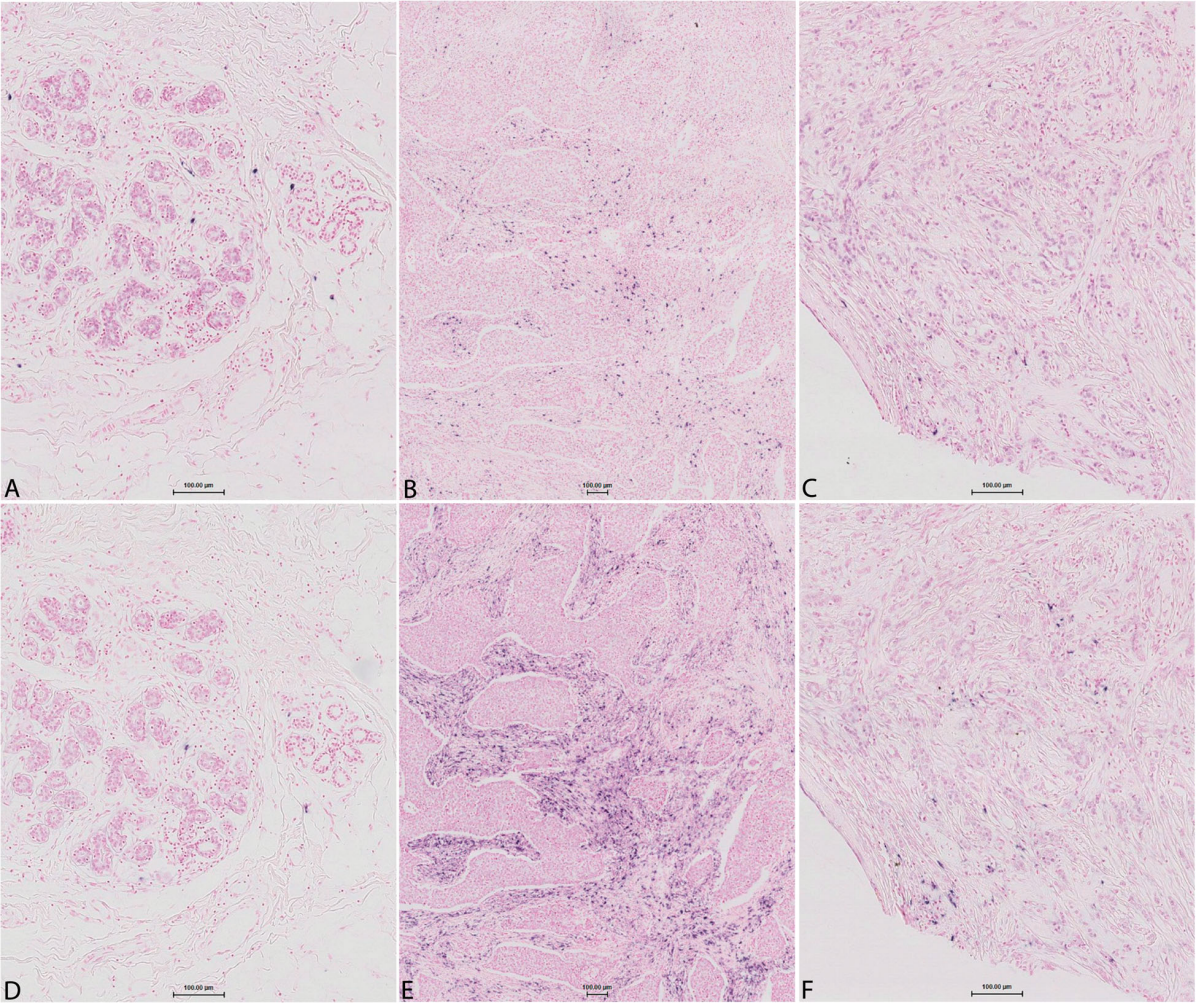
Differential CISH expression levels of LNA 5`-3’DIG hsa (80 nM) probes for miR-18a and miR-18b in ER^+^ vs ER^−^ breast cancers. Top row: miR-18a expression. Bottom row: miR-18b expression. **a**) and **d**) normal epithelial cells in an ER^−^ tumour (sample id: 14 ER^−^ in Table 2). **b**) and **e**) shows an ER^−^ tumour (sample id: 13 ER^−^ in Table 2) with higher expression of miR-18b in comparison with miR-18a. **c**) and **f**) shows an ER^+^ tumour (sample id: 04 ER^+^ in Table 2)

**Fig. 4:**
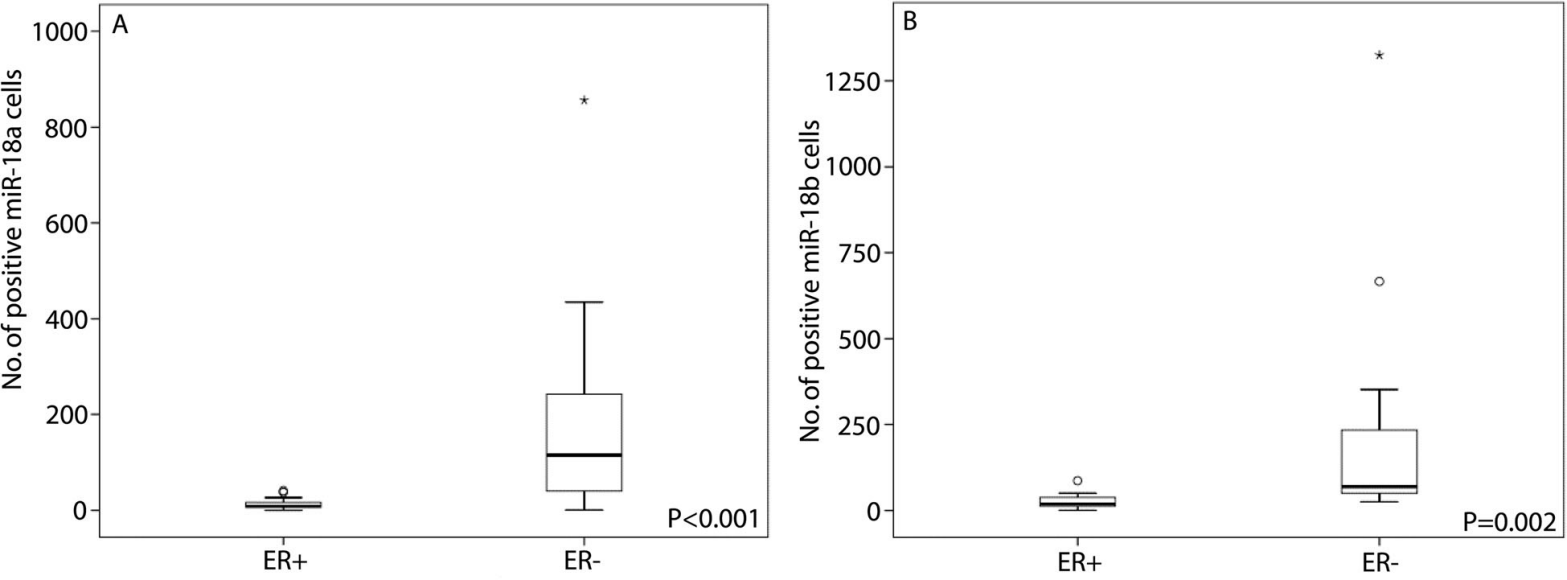
Boxplot of CISH expression quantification, as measured by counting the number of positively stained cells in the *n* = 20 ER^+^ vs the n = 20 ER^−^ breast cancers of **a**) miR-18a and **b**) miR-18b. Central line in boxes represent the median value, boundaries of boxes represent the interquartile range and ends of whiskers represent the minimum and maximum values, excluding outliers. *P*-values were obtained using independent T-test

Detection of miR-18a and miR-18b by CISH in FFPE tissue from breast cancer patients resulted in strong and specific cytoplasmic staining in cells in the intratumoural stroma (Fig. 1) or stroma surrounding the tumour margin (Fig. 2). Both microRNAs were typically found in round shaped cells located within the tumour stroma, although some stained cells were more elongated and outstretched (Fig. 2). Little or no expression was found within the epithelial tumour cells or in cells further (> 0.5 mm) away from the tumour area (Figs. 2 and 3). As expected based on our selection criteria, miR-18a and miR-18b had a significantly lower expression level in ER^+^ tumours in comparison to ER^−^ tumours (Independent T-test, *P* < 0.001 for miR-18a and *P* = 0.002 for miR-18b) (Figs. 3, 4, and Table 2). The expression levels of miR-18a and miR-18b, as measured by CISH-expression levels, showed a strong correlation (Pearson’s correlation coefficient of 0.85 *P* < 0.001). Furthermore, these CISH-expression levels correlated well with those measured by qPCR in our previous study [8], for both miR-18a (r = 0.75 Spearman’s rho test *P* < 0.001) and for miR-18b (r = 0.64, *P* < 0.001).

**Table 2:** Quantification of cells positive for CISH expression of miR-18a and miR-18b

**# / ER-status**	**miR-18a**	**miR-18b**	**# / ER-status**	**miR-18a**	**miR-18b**
01 ER-	13	26	01 ER+	0	1
02 ER-	87	35	02 ER+	11	6
03 ER-	309	41	03 ER+	4	7
04 ER-	11	46	04 ER+	9	10
05 ER-	42	48	05 ER+	9	12
06 ER-	29	54	06 ER+	7	14
07 ER-	82	57	07 ER+	27	15
08 ER-	150	57	08 ER+	8	16
09 ER-	99	60	09 ER+	14	16
10 ER-	211	63	10 ER+	1	18
11 ER-	76	75	11 ER+	9	20
12 ER-	1	83	12 ER+	8	26
13 ER-	39	131	13 ER+	13	31
14 ER-	257	182	14 ER+	19	31
15 ER-	227	211	15 ER+	5	34
16 ER-	435	257	16 ER+	7	43
17 ER-	262	309	17 ER+	14	44
18 ER-	131	352	18 ER+	21	48
19 ER-	209	666	19 ER+	38	51
20 ER-	856	1325	20 ER+	41	86

Quantification of miR-18a and miR-18b expression, visualised by CISH, in 20 ER-positive and 20 ER-negative breast cancer tumours, sorted by miR-18b-expression

### MiR-18a and miR-18b expression pattern in relation to immunohistochemical markers

Although a strong and specific staining method was established, CISH has its limitations. Assessing the CISH slides only, pathologists were in doubt of the cell type that showed positive miR-18a and miR-18b expression. In an attempt to identify the cell type, we performed IHC on serial sections of the same breast tumours, and the corresponding lymph node samples. The following IHC-markers were used: CD4, CD8, CD20, CD68, CD138, PAX5 and actin (Table 1).

**Fig. 5:**
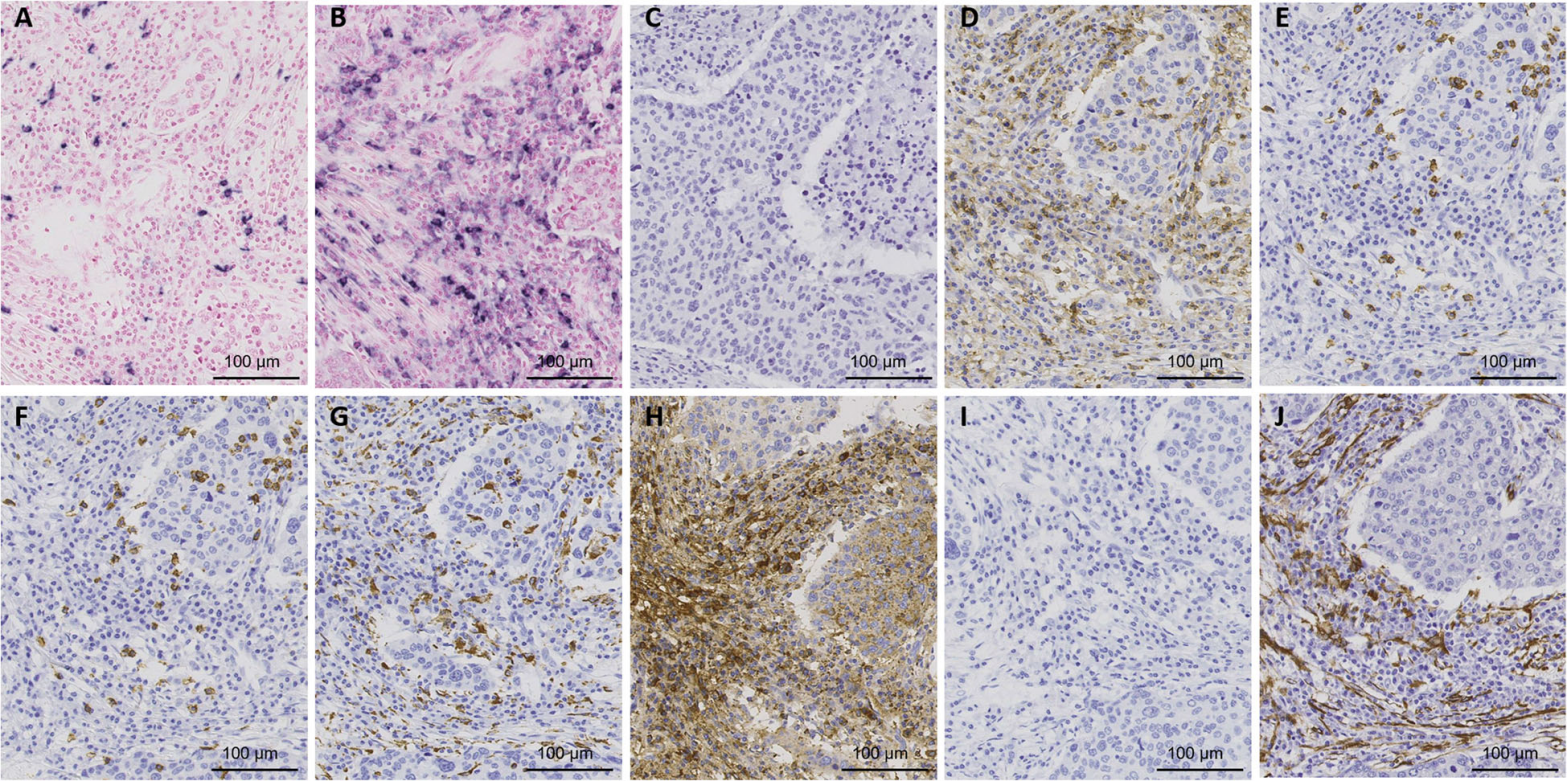
Expression pattern in an ER^−^ breast tumour (sample id: 13 ER^−^ in Table 2) of **a**) CISH LNA 5`-3’DIG hsa (80 nM) miR-18a probe and **b**) CISH LNA 5`-3’DIG hsa miR-18b (80 nM) probe in comparison to IHC staining for **c**) ER, **d**) CD4, **e**) CD8, **f**) CD20, **g**) CD68, **h**) CD138, **i**) PAX5 and **j**) actin. Scale bar 100 μm

A comparison between the different IHC stains and the CISH results for miR-18a and miR-18b showed some overlap with the expression of CD68 (monocytes/macrophages), CD138 (plasma cells) and actin (smooth muscle), although a complete match was not observed (Fig. 5). Actin staining identified fibroblast cells which were mostly oblong and outstretched, and since miR-18a and miR-18b were mainly expressed in round shaped cells, we hypothesized that the miR-18a and miR-18b positive cells are more likely to be associated with cells of lymphoid or myeloid origin.

**Fig. 6:**
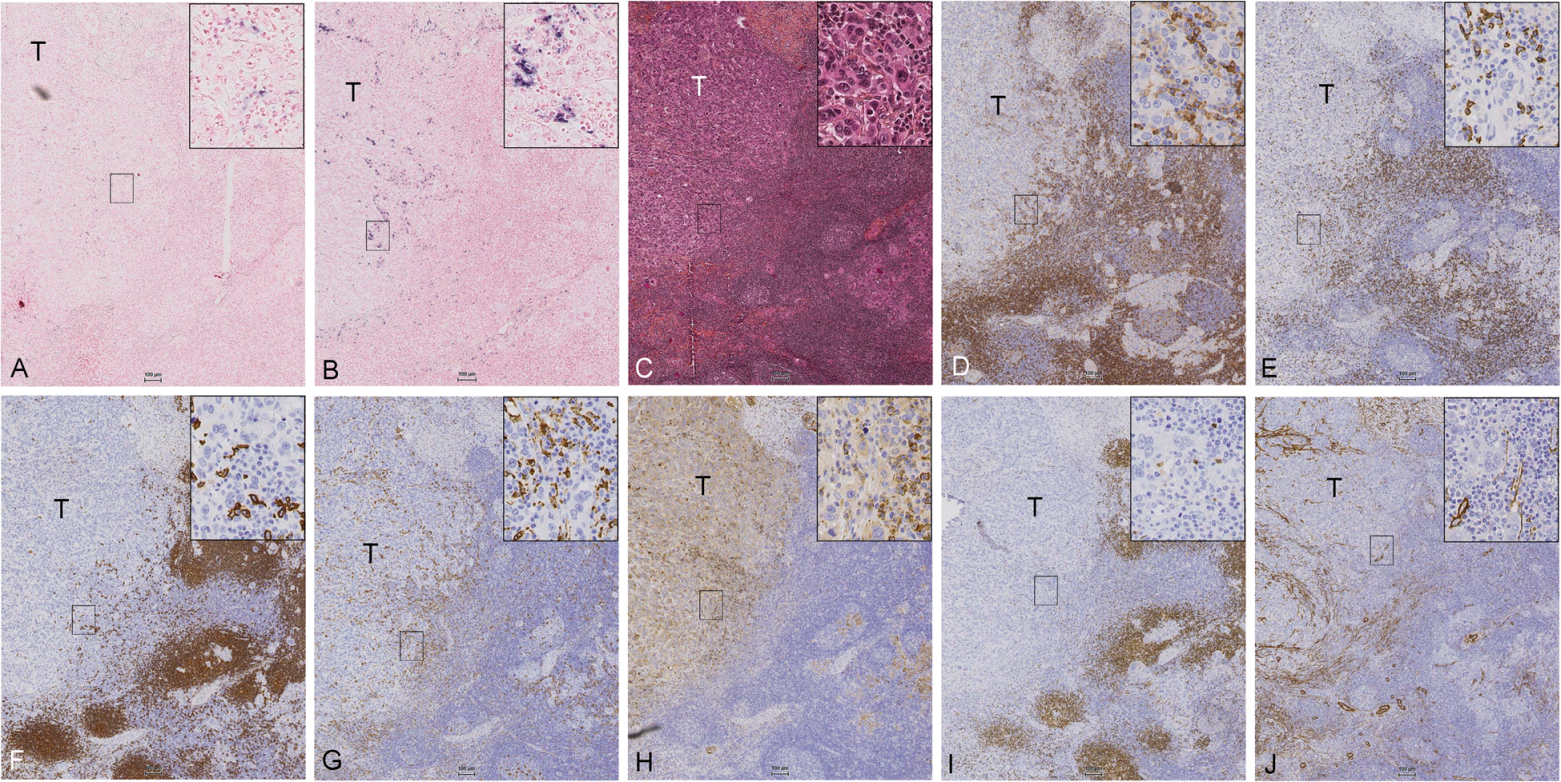
Expression pattern comparison in a lymph node with tumour infiltration from a patient with an ER^−^ breast tumour. **a**) CISH LNA 5`-3’DIG hsa (80 nM) miR-18a probe and **b**) CISH LNA 5`-3’DIG hsa (80 nM) miR-18b probe, in comparison to IHC staining for **c**) HE, and IHC-staining for **d**) CD4, **e**) CD8, **f**) CD20, **g**) CD68, **h**) CD138, **i**) PAX5, and **j**) actin. **T** indicates tumour area. Scale bar 100 μm

Based on this we also stained lymph nodes from breast cancer patients both positive and negative for cancer cells (based upon histology from HE-slides), for the same IHC-markers as mentioned above, as well as for miR-18a and miR-18b. In the lymph nodes containing tumour cells, all the miR-18a- and miR-18b-positive cells were localized close to or in between the tumour cells (Fig. 6), thus following the pattern we observed in the primary tumours (for comparison with corresponding primary tumour, see Additional file 3, S1 Fig). This seemed especially true in patients with ER^−^ tumours (ER^−^ tumour in Fig. 6, an ER^+^ tumour in Additional file 4, S2 Fig). For the IHC markers, only the expression patterns for CD68 and CD138 showed some similarity with miR-18a and miR-18b expression, both in location and the shape and size of the positively stained cells (Fig. 6). Lymph nodes from breast cancer patients without tumour cells had a much more scattered staining pattern for miR-18a and miR-18b, with few positive cells and more stained cells in germinal centres. These results suggest that the miR-18a and miR-18b-expressing cells could be part of an immune response directed towards the tumour, and more specifically towards ER^−^ tumour cells.

**Fig. 7:**
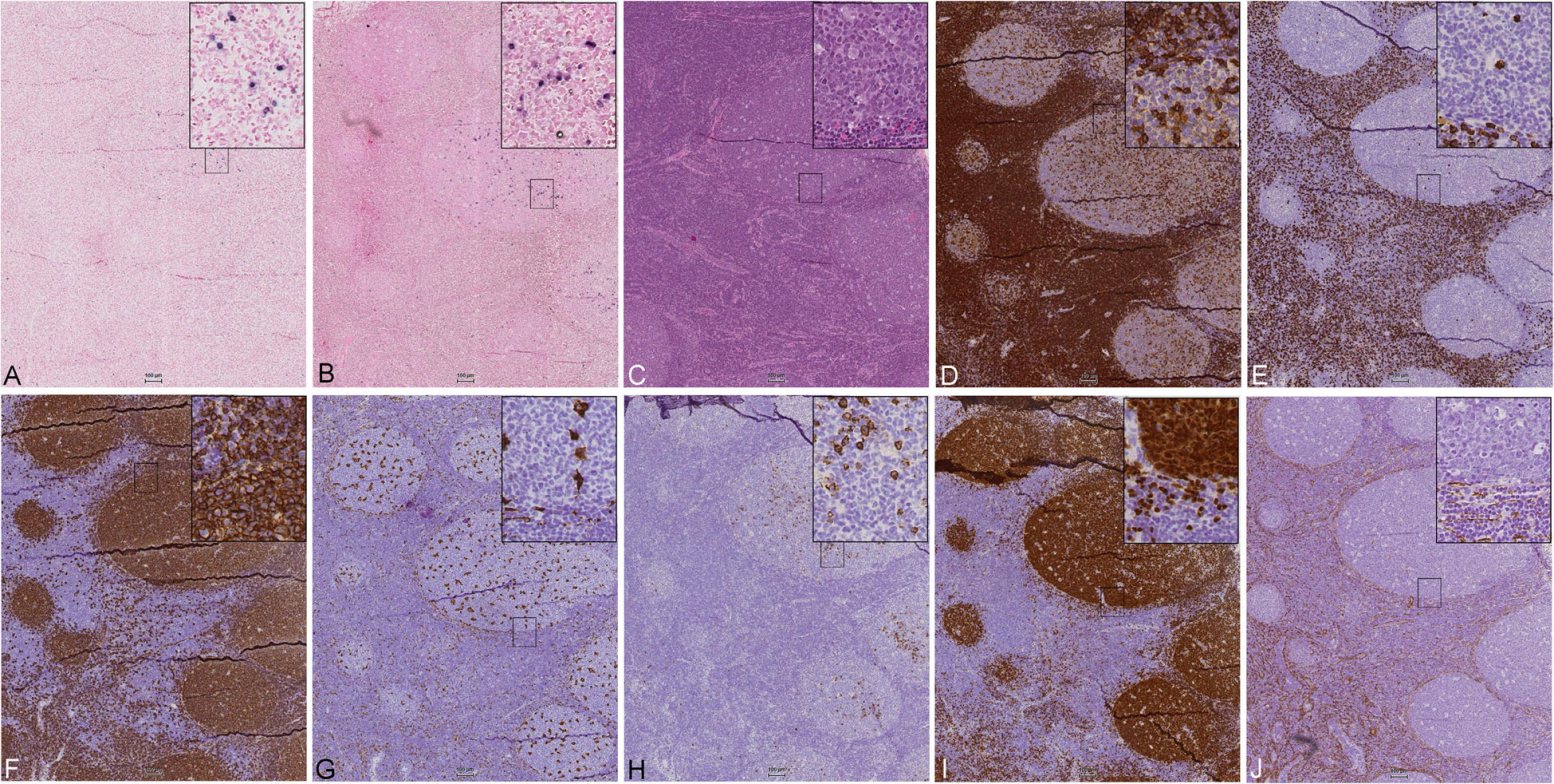
Expression pattern comparison in a benign lymph node diagnosed as reactive lymphadenitis from the neck. **a**) CISH LNA 5`-3’DIG hsa (80 nM) miR-18a probe and **b**) CISH LNA 5`-3’DIG hsa (80 nM) miR-18b probe, in comparison to IHC staining for **c**) HE, and IHC-staining for **d**) CD4, **e**) CD8, **f**) CD20, **g**) CD68, **h**) CD138, **i**) PAX5, and **j**) actin. Scale bar 100 μm

To investigate whether this reaction was cancer specific, we also analysed lymph nodes from patients with non-malignant disease, here in a case of reactive lymphadenitis (Fig. 7). In these non-malignant reactive lymph nodes miR-18a and -18b staining was mostly observed in the germinal centres. This, was in contrast with our observation in lymph nodes containing cancer cells where no staining was observed in the germinal centres. Again, comparison with the staining patterns for the IHC markers showed only partial overlap with CD68 and CD138 (Fig. 7).

### MiR-18a and miR-18b expression pattern in relation to tumour infiltrating lymphocytes

**Fig. 8:**
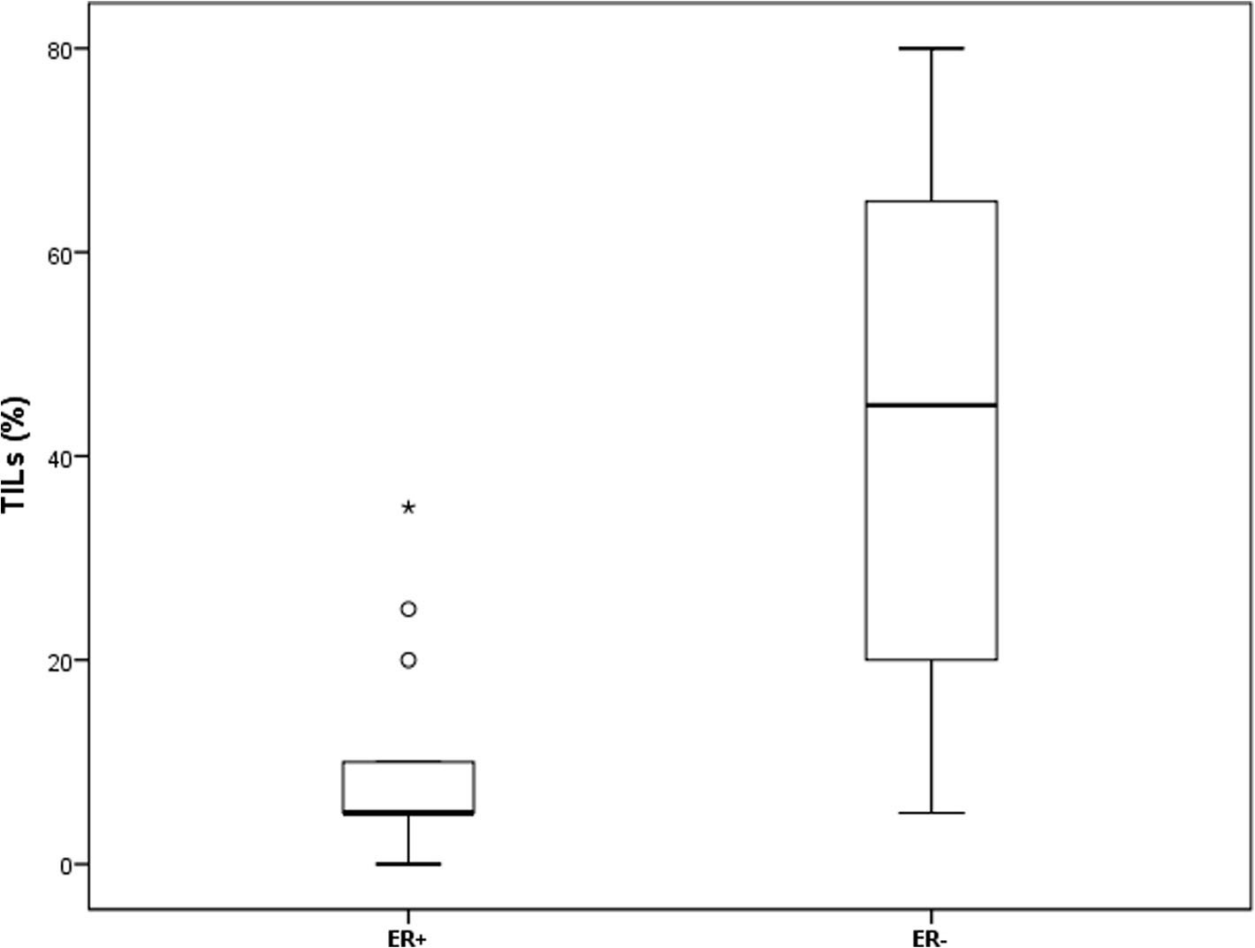
Boxplot of TILs quantification, as measured by scoring the % of tumor-infiltrating lymphocytes in the n = 20 ER^+^ vs the n = 20 ER^−^ breast cancers in the CISH-cohort. Central line in boxes represent the median value, boundaries of boxes represent the interquartile range and ends of whiskers represent the minimum and maximum values, excluding outliers

Measurement of TILs in the 40 ER^+^ and ER^−^ tumour tissues showed that the ER^−^ tumours had a significantly higher number of TILs (Independent T-test, *P* = 0.0001, boxplot in Fig. 8). As such we analysed TILs in 204 samples from our previous study [8], and compared it with the expression of miR-18a and -18b (measured by qPCR). Patients were also grouped based on ER status and high vs low TILs (Additional file 5, S3 Fig). Although not significant, a difference was observed between ER^−^/high TILs versus ER^−^/low TILs for both microRNAs. Additionally, the ER^−^ with high TILs had significantly (*P* < 0.001) higher expression of miR-18a and miR-18b, than the ER^+^ patients with high TILs (see boxplot in Additional file 5, S3 Fig). Again, this suggests that miR-18a and miR-18b expression is related to TILs and ER^−^ cancers. Furthermore, miR-18a and -18b expression was also found in the stroma of both pancreatic cancer, and lung cancer tissue (Additional file 6, S4 Fig).

### MiR-18a and miR-18b expression and CIBERSORT

To address the heterogeneity of immune cells in bulk tumour tissue, and to further investigate which type of cells express miR-18a and/or miR-18b, we used CIBERSORT [30] to characterize the cell composition of the bulk tumour tissue from which mRNA and miRNA was isolated. The output from CIBERSORT is a matrix of quantification levels of each of 22 immune cell types for each tumour. For each cell type, we took the vector of relative or absolute levels for all tumours and then compared this with the expression of miR-18a and miR-18b for the same tumours. The resulting Spearman’s Rho correlation coefficients are shown in the attached Table 3. For both relative and absolute assessment, the “M1 macrophages” cell type had the highest and most significant correlation in the Stavanger data. The cell type with the second highest correlation was “memory activated CD4+ T-cells”. For the Oslo2 cohort, the latter cell type showed the highest positive correlation and the “M1 macrophages” showed the second highest correlation (Table 3).

**Table 3:** CIBERSORT analyses, Stavanger array cohort and Oslo2 cohort

**Stavanger array cohort (*n*** **= 94)**					**Oslo2 cohort (*n*** **= 377)**				
	hsa-miR-18a		hsa-miR-18b			hsa-miR-18a		hsa-miR-18b	
	Corr.^a^	*P*	Corr.^a^	*P*		Corr.^a^	*P*	Corr.^a^	*P*
M1 Macrophages	0.415	0.001	0.387	0.001	Memory activated CD4+ T-cells	0.265	0.001	0.303	0.001
Memory activated CD4+ T-cells	0.328	0.001	0.308	0.002	Activated Dendritic cells	0.245	0.001	0.242	0.001
M0 Macrophages	0.299	0.003	0.279	0.006	M1 Macrophages	0.241	0.001	0.286	0.001
Monocytes	0.159	0.125	0.157	0.132	Neutrophils	0.177	0.001	0.151	0.003

List of the top four immune cells that correlate with miR-18a and miR-18b expression. ^a^ indicates Absolute Spearman’s Rho correlation

## Discussion

High expression of miR-18a and miR-18b is known to be associated with ER^−^ breast cancer, high proliferation and worse prognosis [8, 39]. However, the role and function of these two microRNAs is not well understood. Our current in situ localisation shows that miR-18a and miR-18b are specifically expressed in the intratumoural stroma and in the stroma directly surrounding ER^−^ tumours with a high degree of TILs. Additionally, the current study demonstrates the specificity of our CISH protocol, and subsequently confirms our previous qPCR results that miR-18a and miR-18b are highly expressed in ER^−^, and low in ER^+^ breast cancers [8, 9].

Few studies have evaluated miR-18a or miR-18b expression patterns in cancer tissue using CISH, and only Guo et al. [40] showed that miR-18a is expressed in the tumour cells of ER^+^ breast cancer tissue. These authors also describe that miR-18a is significantly under-expressed in ER^−^ breast cancers, this is in contrast to most studies that report higher expression in ER^−^ breast cancers. There are some technical differences in the CISH protocol between Guo et al. and the current study, and this might explain the differences in level of expression and location. The localisation of miR-18a and miR-18b expression is important in order to understand the role of these microRNAs in ER^−^ breast tumours and in breast cancer progression.

The use of CISH in the current study clearly shows that the expression of miR-18a and miR-18b is located in the stroma of ER^−^ breast cancer with a high number of TILs. Furthermore, in breast cancer patients these microRNAs are also observed in lymph nodes both with and without macroscopically confirmed tumour cells. In lymph nodes from patients without cancer, the miR-18a and -18b positive cells were found only in the germinal centres. Meanwhile, in metastatic lymph nodes of breast cancer patients, miR-18a and -18b positive cells were found close to the tumour cells, and absent in the germinal centres. These observational results should be considered preliminary, nonetheless they do suggest a potential migration or activation of specific immune cells, related to ER^−^ breast tumour cells.

In accordance with the stromal localization of miR-18a and -18b positive cells demonstrated by CISH and the partial overlap with CD68 staining, CIBERSORT analyses in two different cohorts showed a significant positive correlation between M1 / M0 macrophages and the expression of miR-18a and miR-18b. Similarly, Halvorsen et al. showed a correlation between the miR17 ~ 92 cluster and CD68 positive cells (i.e. monocytes/macrophages) when investigating microRNAs isolated from the tumour interstitial fluid from breast cancer patients [21].

Interestingly, together with several other microRNAs, miR-18b has been suggested to play an important part in macrophage lineage development, through regulation of important macrophage transcription factors such as PU.1, RUNX1, CSFR1, PPARα and PPARγ [41]. As such, one might stipulate that overexpression of miR-18b might lead to increased expression of cytokines such as IL1β, IL-6 and TNFα, thereby increasing a pro-inflammatory condition in the TME. Additionally, miR-18a-5p can promote carcinogenesis by directly targeting interferon regulatory factor 2 (IRF2) [42]. IRF2 is a member of the IRF family, which has the ability to exert anti-oncogenic activities; others showed that IRF2 is an important regulator of the pro-inflammatory response in macrophages by controlling HIF-1α–dependent glycolytic gene expression and glycolysis [43]. Interestingly, miR-18a has also been identified as an upstream regulator of hypoxia-inducible factor 1α (HIF1A) [44, 45]. HIF1A is associated with macrophage function, whereby its overexpression induces macrophage M1 polarization [46], and it also plays a role in centrosome aberrations and tumour progression in TNP breast cancer [47].

CIBERSORT analyses in two independent cohorts resulted in a positive miR-18a and -18b correlation with CD4+ T-cell memory cells; a subset of T-cells that can recognize foreign invaders such as bacteria or viruses, as well as cancer cells. Vahidi et al. [48] recently studied different subtypes of memory T-cells in the CD4+ population in tumour draining lymph nodes of 52 untreated breast cancer patients. Among all the CD4+ memory T-cells, more than 70% of the cells exhibited a memory phenotype, and in the tumour positive lymph nodes the frequency of T stem cell memory cells was higher than in tumour negative lymph nodes [48]. Jiang et al. studied the role of the miR-17 ~ 92 cluster during the T-cell antigen response and showed that miR-18a counteracts other microRNAs (e.g. miR-17 and miR-19 display a pro-Th1 function) by inhibition of proliferation and an increase in activation-induced cell death of CD4+ T-cells [49].

Both we and others have shown that miR-18a and miR-18b are related to ER^−^ tumours, and several studies have shown that both miR-18a and miR-18b directly repress ER activity [13–15, 17, 50, 51] and thus direct the location of these microRNA to the cancer cells.

It has also been demonstrated that microRNAs have the ability to take part in crosstalk between tumour cells and the microenvironment, by exosomal delivery [25, 26]. From different cancer studies circulating miR-18a has been detected as a potential microRNA biomarker for early detection of cancer in serum samples [52, 53]. Meanwhile, analysis of serum samples from 60 breast cancer patients with triple-negative tumours showed that miR-18b has prognostic value for distant metastases and overall survival [39]. These results show that miR-18a and -18b could be detected in liquid biopsies, and might therefore be potential biomarkers for tumour progression and worse prognosis in breast cancer.

The IHC staining performed and presented in the current study does not show a complete overlap with miR-18a and miR-18b (Table 1). Still, the finding of expression of these microRNAs in both elongated and smaller round cells could fit with expression in both macrophages and T-cells. A combination of both IHC and CISH on the same slide, or staining with several antibodies simultaneously, might be an appropriate way to improve the identification of the proper cell type(s) expressing these microRNAs.

Based on existing literature (cited above) and our studies, we can only conclude that miR-18a and miR-18b appears to be highly expressed among TILs in ER^−^ breast cancer, and that the expression of these microRNAs is correlated with a worse prognosis in these patients. While miR-18a and -18b might be linked to macrophages and memory T-cells, we speculate that these cells are not effective enough to stop the tumours from forming metastases.

There are some limitations to this study; first of all, we have only evaluated 40 patients with the CISH method. Second, the in situ expression of miR-18a and -18b did not show a complete overlap with any of the IHC markers. Third, although the CIBERSORT results were significant in two independent cohorts, this data shows only an association between the miR-18a and miR-18b expression and the different immune cells. These results should therefore be interpreted with caution. The exact function of these microRNAs in breast cancer stromal tissue, what type of cells express them, and how they relate to the infiltration of immune cells such as macrophages, needs further investigation.

## Conclusions

In conclusion, our results show that miR-18a and miR-18b are highly expressed in the stromal compartment adjacent to ER^−^ tumour cells, especially in areas containing a high degree of infiltrating lymphocytes. The expression of miR-18a and miR-18b is positively correlated with the presence of macrophages and CD4 memory T-cells. We hypothesize that the expression of these microRNAs is related to a systemic immunological response, possibly produced by monocytes/macrophages that are activated in lymph nodes, and thereafter homed towards specific tumours. Further investigation in larger patient cohorts is needed to validate these miR-18a and miR-18b-expressing stromal cells as macrophages.

## Supplementary information

**Additional file 1: S1 Table.** Patient characteristics in the CISH cohort.

**Additional File 2: S2 Table.** Name, sequence, RNA Tm and concentration for the LNA™ 5`-3’DIG hsa detection probes (Exiqon) used in CISH experiments.

**Additional file 3: S1 Fig.** miR-18b expression in primary tumour corresponding to Fig. 6. Positive and specific CISH expression of LNA 5`-3’DIG miR-18b (80 nM) in the stroma of a representative lymph node-positive primary breast cancer tumour.

**Additional file 4: S2 Fig**. Expression pattern comparison in a lymph node with tumour infiltration from a patient with an ER^+^ breast tumour. A) CISH LNA 5`-3’DIG hsa (80 nM) miR-18a probe and B) CISH LNA 5`-3’DIG hsa (80 nM) miR-18b probe, in comparison to IHC staining for C) HE, and IHC-staining for D) CD4, E) CD8, F) CD20, G) CD68, H) CD138, I) PAX5, and J) actin.

**Additional file 5: S3 Fig.** Expression measured with qPCR in ER^+^ and ER^−^ breast cancers with high and low TILs of A) miR-18a and B) miR-18b. Central line in boxes represent the median value, boundaries of boxes represent the interquartile range and ends of whiskers represent the minimum and maximum values, excluding outlies. *P*-values were obtained using independent T-test.

**Additional file 6: S4 Fig.** CISH expression demonstrating strong and specific positive staining with LNA 5`-3’DIG hsa (80 nM) miR-18b probe expression in A) pancreatic cancer, and B) lung cancer.

## Data Availability

The mRNA dataset for the Stavanger array cohort is publicly available at the online Gene Expression Omnibus (GEO) repository: accession number GSE46563. The Oslo2 microRNA expression data are available from the GEO repository with accession number GSE58210, while the mRNA expression data has accession number GSE58212. Additional datasets used and/or analysed during the current study are available from the corresponding author on reasonable request.

## Abbreviations

ER: Oestrogen receptor alpha
TME: Tumour microenvironment
FFPE: Formalin fixed paraffin embedded
CISH: Chromogenic in situ hybridization
TILs: Tumour infiltrating lymphocytes
TAM: Tumour associated macrophages
IHC: Immunohistochemistry
qPCR: Quantitative real-time PCR
PR: Progesterone receptor
HER2: Human epidermal growth factor receptor 2
TNP: Triple-negative phenotype
