# Supplementary material for: MiR-18a and miR-18b are expressed in the stroma of oestrogen receptor alpha negative breast cancers
Source: BMC Cancer. 2020 May 5;20:377. doi: 10.1186/s12885-020-06857-7 (PMC7201801; doi:10.1186/s12885-020-06857-7)
Supplement: Supplementary file 1 — Additional file 1: S1 Table. Patient characteristics in the CISH cohort. [file 12885_2020_6857_MOESM1_ESM.docx]

**S1 Table.** Patient characteristics in the CISH cohort.

| **Patient characteristic** | **Frequencies n=40** |
| --- | --- |
| Age |  |
| <55 years | 23 |
| ≥55 years | 17 |
| Nottingham grade |  |
| 1 | 13 |
| 2 | 9 |
| 3 | 18 |
| Tumour size |  |
| <2 cm | 25 |
| ≥2 cm | 15 |
| ER |  |
| Positive | 20 |
| Negative | 20 |
| PR |  |
| Positive | 15 |
| Negative | 25 |
| HER2* |  |
| Positive | 2 |
| negative | 37 |
| TNP |  |
| Positive | 23 |
| Negative | 17 |

*HER2 missing for 1 patient
