# Supplementary material for: MiR-18a and miR-18b are expressed in the stroma of oestrogen receptor alpha negative breast cancers
Source: BMC Cancer. 2020 May 5;20:377. doi: 10.1186/s12885-020-06857-7 (PMC7201801; doi:10.1186/s12885-020-06857-7)
Supplement: Supplementary file 2 — Additional File 2: S2 Table. Name, sequence, RNA Tm and concentration for the LNA™ 5`-3’DIG hsa detection probes (Exiqon) used in CISH experiments. [file 12885_2020_6857_MOESM2_ESM.docx]

**S2 Table.** Name, sequence, RNA Tm and concentration for the LNA™ 5`-3`DIG hsa detection probes (Exiqon) used in CISH experiments.

| LNA^TM^ Detection/control probes | Sequence | RNA Tm calc. | Concentration |
| --- | --- | --- | --- |
| 5`-3`DIG hsa miR-18a | CTA**T**CTGCACTAGATGCACCTTA | 88 °c | 80 nM |
| 5`-3`DIG hsa miR-18b | CTA**A**CTGCACTAGATGCACCTTA | 89 °c | 80 nM |
| 5`-DIG U6 snRNA | CACGAATTTGCGTGTCATCCTT | 84 °C | 2.0 nM |
| 5`-DIG Scrambled | GTGTAACACGTCTATACGCCCA | 87˚C | 80 nM |

Note: the sequences for miR-18a and miR-18b differ by only one nucleotide, as indicated by **T** and **A**.
