# Supplementary figures and images for: MiR-18a and miR-18b are expressed in the stroma of oestrogen receptor alpha negative breast cancers
Source: BMC Cancer. 2020 May 5;20:377. doi: 10.1186/s12885-020-06857-7 (PMC7201801; doi:10.1186/s12885-020-06857-7)

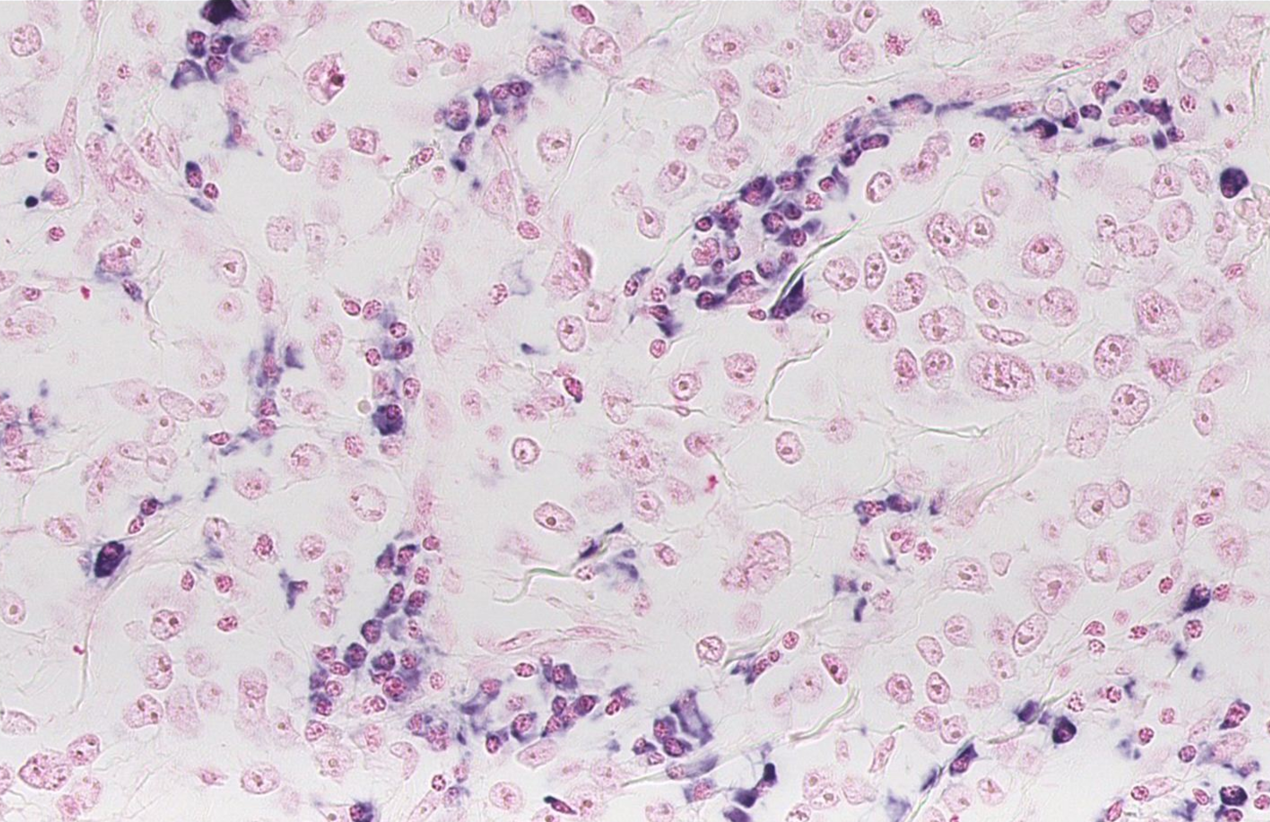

Supplement: Supplementary file 3 — Additional file 3: S1 Fig. miR-18b expression in primary tumour corresponding to Fig. 6. Positive and specific CISH expression of LNA 5`-3’DIG miR-18b (80 nM) in the stroma of a representative lymph node-positive primary breast cancer tumour. [file 12885_2020_6857_MOESM3_ESM.tif]

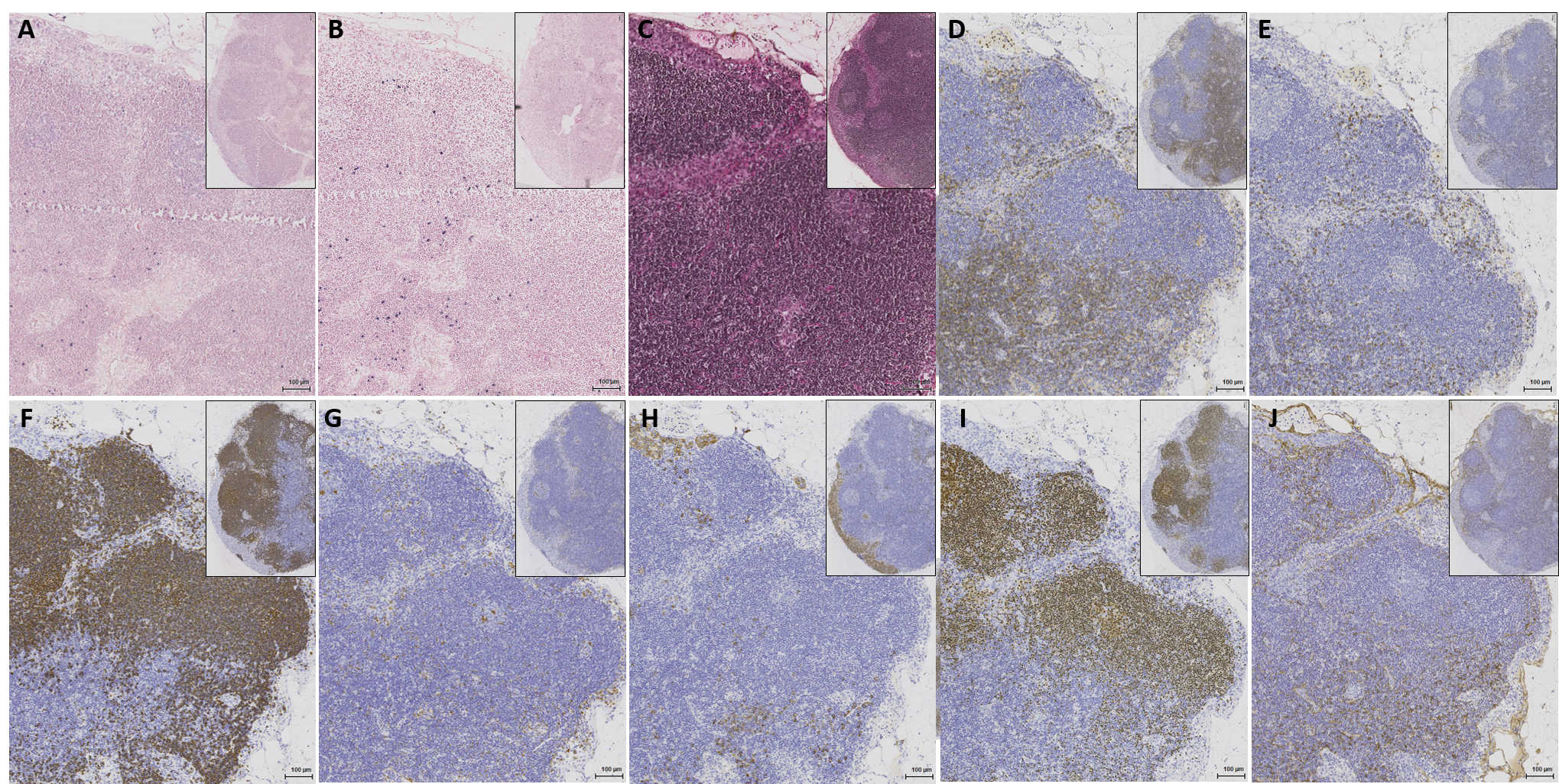

Supplement: Supplementary file 4 — Additional file 4: S2 Fig. Expression pattern comparison in a lymph node with tumour infiltration from a patient with an ER+ breast tumour. A) CISH LNA 5`-3’DIG hsa (80 nM) miR-18a probe and B) CISH LNA 5`-3’DIG hsa (80 nM) miR-18b probe, in comparison to IHC staining for C) HE, and IHC-staining for D) CD4, E) CD8, F) CD20, G) CD68, H) CD138, I) PAX5, and J) actin. [file 12885_2020_6857_MOESM4_ESM.png]

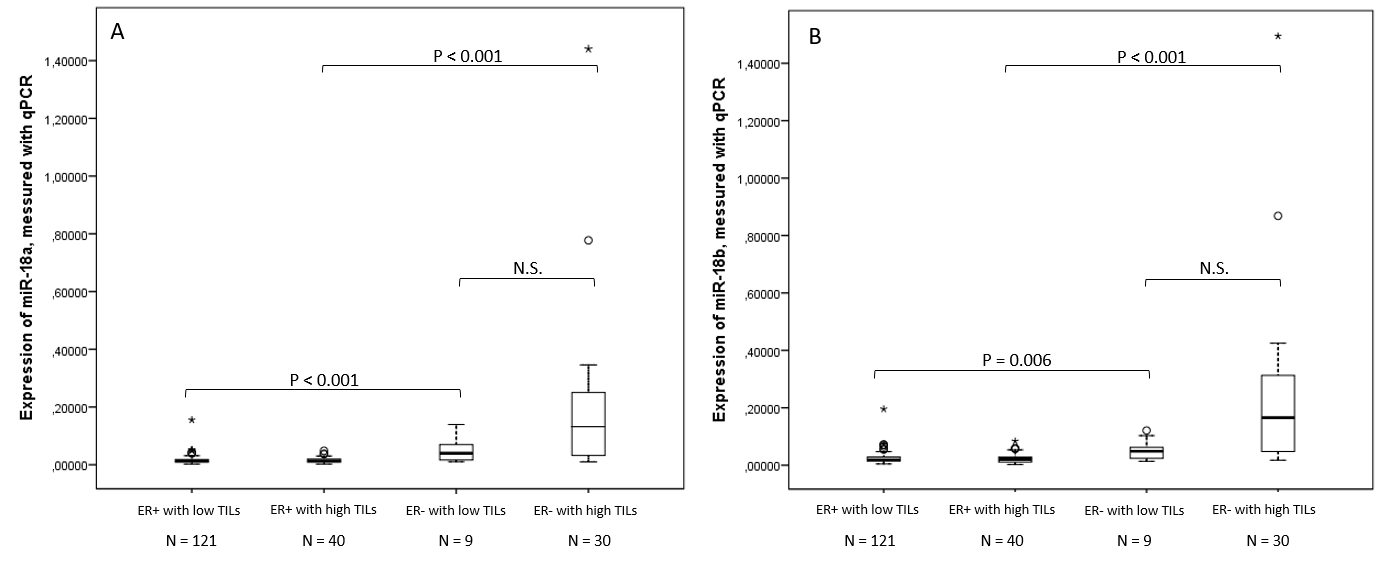

Supplement: Supplementary file 5 — Additional file 5: S3 Fig. Expression measured with qPCR in ER+ and ER− breast cancers with high and low TILs of A) miR-18a and B) miR-18b. Central line in boxes represent the median value, boundaries of boxes represent the interquartile range and ends of whiskers represent the minimum and maximum values, excluding outlies. P-values were obtained using independent T-test. [file 12885_2020_6857_MOESM5_ESM.png]

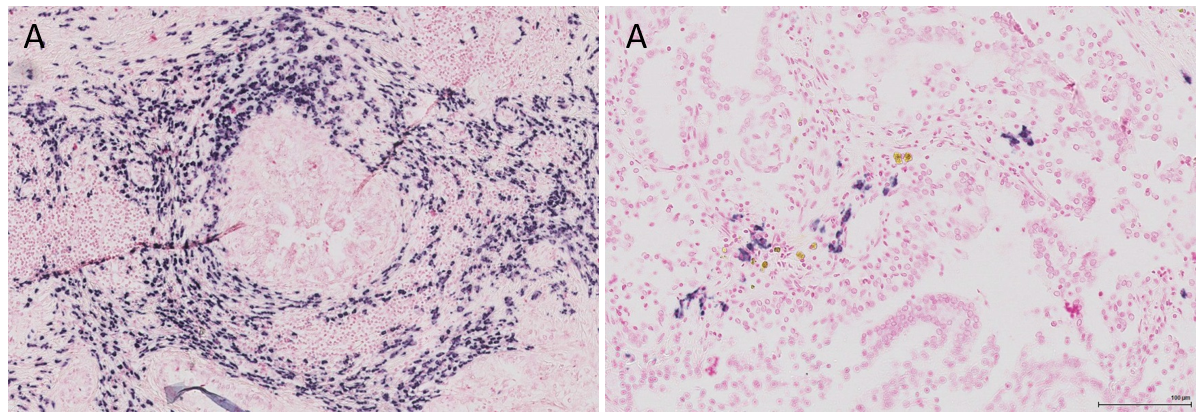

Supplement: Supplementary file 6 — Additional file 6: S4 Fig. CISH expression demonstrating strong and specific positive staining with LNA 5`-3’DIG hsa (80 nM) miR-18b probe expression in A) pancreatic cancer, and B) lung cancer. [file 12885_2020_6857_MOESM6_ESM.tif]
